# Coupling Dysfunction of Mitochondria and Actin in Loss of Native Gastric Glands: A Mechanistic Hypothesis for Chronic Atrophic Gastritis Pathogenesis

**DOI:** 10.3390/cells15181670

**Published:** 2026-09-15

**Authors:** Wenxin Wei, Yue Ma, Shujie Wang, Cisong Cheng

**Affiliations:** School of Basic Medical Sciences, Chengdu University of Traditional Chinese Medicine, Chengdu 611137, China; 13693446802@163.com (W.W.);

**Keywords:** chronic atrophic gastritis, actin cytoskeleton, mitochondrial damage, glandular atrophy, inflammation-driven carcinogenesis

## Abstract

**Highlights:**

**What are the main findings?**

- Oxidative stress and mitochondrial ATP depletion synergistically dismantle the gastric actin cytoskeleton, leading to structural and functional glandular degradation.
- ROS-induced chemical damage to actin and aberrant cofilin activation uncouple the energy cytoskeleton axis, triggering a vicious cycle of epithelial degeneration.

**What are the implications of the main findings?**

- The destabilization of the actin network is proposed as a potential structural driver contributing to the progressive loss of native gastric glands in chronic atrophic gastritis (CAG).
- Restoring the homeostatic coupling between mitochondrial bioenergetics and cytoskeletal dynamics offers a novel therapeutic strategy to halt inflammation-driven carcinogenesis.

**Abstract:**

The progressive loss of native gastric glands in chronic atrophic gastritis (CAG) represents a critical step toward gastric carcinogenesis, yet the underlying cellular structural mechanisms remain incompletely understood. In this review, we formulate an integrative mechanistic hypothesis that disruption of actin cytoskeletal homeostasis, driven by mitochondrial bioenergetic dysfunction, plays a central role in the progressive loss of native gastric glands. Persistent *H. pylori* infection and chronic mucosal inflammation may initiate a self-reinforcing cycle of metabolic and structural impairment: mitochondrial ATP depletion compromises the energy supply required for F-actin polymerization, whereas oxidative stress promotes aberrant activation of the cofilin pathway, resulting in increased filament disassembly. This imbalance in the energy–cytoskeleton axis may impair epithelial polarity signaling and mechanotransduction, thereby contributing to stem cell dysregulation and progressive mucosal degeneration. While this synthesis relies primarily on in vitro and multi-omics evidence, it provides a complementary perspective on CAG pathogenesis and highlights potential therapeutic strategies aimed at restoring cellular mechanical and metabolic homeostasis to prevent precancerous progression.

## 1. Introduction

Chronic atrophic gastritis (CAG) is a well-recognized precancerous condition in gastric carcinogenesis characterized by the progressive loss of native gastric glands (with or without metaplastic replacement). Although autoimmune CAG is relatively rare [1], *H. pylori*-associated atrophic gastritis remains highly prevalent in East Asia and other high-risk regions and poses a significant clinical burden [2,3]. Emerging evidence suggests that, beyond chronic inflammation, alterations in cytoskeletal organization play important roles in CAG progression. As a fundamental structural framework maintaining epithelial polarity and mechanical barrier integrity, the actin cytoskeleton is increasingly recognized as a key regulator of epithelial homeostasis. Its destabilization is proposed to contribute to glandular disintegration and inflammation-associated carcinogenesis [4]. Currently, effective therapies for reversing the loss of native gastric glands are limited. Although various therapeutic strategies, including the use of bioactive compounds, have shown potential in delaying inflammation-driven carcinogenesis, their underlying regulatory mechanisms—particularly those involving cytoskeletal organization and associated signaling networks—remain incompletely understood [5]. In this review, we aim to provide a complementary perspective on CAG pathogenesis by integrating cytoskeletal dynamics with epithelial polarity and mechanotransduction signaling. Furthermore, we explore putative pharmacological directions for restoring “energy–cytoskeleton” coupling and cellular homeostasis, aiming to delay or halt disease progression along the precancerous Correa cascade [6].

## 2. Structural Basis for Gastric Gland Polarity Establishment and Secretory Function: The Actin Cytoskeleton

Based on established generalized epithelial models, the actin cytoskeleton is recognized not only as a physical scaffold supporting cellular morphology but also as a central signaling platform regulating epithelial polarity and intercellular junctions [7,8]. We propose that these fundamental structural principles are equally foundational for maintaining the complex architecture and secretory activity of native gastric glands. Under healthy physiological conditions, F-actin dynamically polymerizes to form cortical rings that precisely anchor tight junction proteins (such as ZO-1) and adherens junction complexes. This dynamic assembly constructs a selectively permeable barrier with mechanical resilience [9,10]. In gastric epithelial and parietal cell physiology [11], this structural integrity is proposed to serve as the primary line of defense against intraluminal acid erosion and prevent the penetration of inflammatory mediators.

Furthermore, actin cytoskeleton remodeling orchestrates the precise regulation of glandular secretion. In generalized epithelial models, actin dynamics and associated proteins are fundamental for polarizing vesicle trajectories and maintaining apical domain integrity [12]. Specifically within the gastric mucosa, these cytoskeletal networks orchestrate the highly polarized and efficient secretion of gastric acid and pepsinogen by promoting vesicle fusion with the apical membrane of parietal and chief cells [13]. Additionally, within the proliferative zone of the glandular neck, mechanochemical coupling mediated by cytoskeletal tension is hypothesized to regulate the orderly proliferation, differentiation, and migration of stem/progenitor cells, as well as the timely apoptotic clearance of senescent cells, a homeostatic process governing gastric cellular plasticity and mucosal renewal [14,15,16,17].

Notably, barrier maintenance, vesicle transport, and stem cell renewal are all active remodeling processes that are highly dependent on the ATP supply [18]. This intimate coupling between structure and energy maintains the structural and functional integrity of the gastric glands. Once homeostasis is disrupted, a vicious cycle of energy metabolism dysfunction and cytoskeletal disintegration is hypothesized to be initiated, potentially contributing to the progressive loss of native gastric glands.

## 3. Pathological Mechanisms of Actin Cytoskeletal Dysregulation: From Exogenous Insults to Endogenous Collapse

### 3.1. Exogenous Initiating Factors: H. pylori–Induced Perturbation and the Inflammatory Microenvironment

The development of CAG arises from persistent pathological alterations in the gastric mucosal microenvironment. While experimental models of intestinal organoids suggest that short-term or controlled inflammatory stimuli may induce adaptive repair responses by modulating stem cell activity and barrier function [19], and similar defensive adaptations are observed in the gastric mucosa [20], this equilibrium is disrupted under persistent *H. pylori* infection, which is proposed to trigger cytoskeletal disruption and eventual progressive loss of native gastric glands.

#### 3.1.1. Upstream Signaling Transduction: SRC Kinase Activation and the Rho/ROCK Axis

Signal transduction mediated by Rho family GTPases plays a central role in maintaining epithelial cytoskeletal tension, intercellular junctions, and tissue integrity. Under *H. pylori* infection and exposure to proinflammatory cytokines such as TNF-α/IL-6, the RhoA/ROCK signaling axis is aberrantly activated, which is a characteristic feature demonstrated in gastric cancer models [21] and is hypothesized to be initiated during early precancerous stages. This process is suggested to inhibit the normal depolymerization of F-actin while disrupting tight junctions. Importantly, cortactin is not a direct downstream target of RhoA; instead, cytoskeletal and junctional disruption is driven by parallel pathogen-hijacked signaling networks, particularly the focal adhesion kinase (FAK) and SRC family kinase axis [22]. Consequently, the loss of gastric mucosal barrier function potentially promotes the progression of chronic gastritis toward oncogenic transformation [23,24].

Based on established in vitro cellular infection models, upon bacterial adhesion, the translocated *H. pylori* virulence factor CagA—which lacks intrinsic kinase activity—is rapidly phosphorylated by host tyrosine kinases, predominantly members of the SRC and Abl families [22]. Drawing insights from broader epithelial remodeling paradigms, we hypothesize that this SRC-dependent cascade serves as a proximal trigger that subverts host adaptor proteins to drive cytoskeletal reorganization during the progressive stages of chronic gastric injury [25]. Subsequently, CagA elicits the “hummingbird phenotype” via the Abl/Rac1 axis, resulting in the loss of epithelial cell polarity and cell scattering [4,26,27]. This morphological transition marks the initiation of a pathological cascade driving the malignant transformation of epithelial cells [26]. Rather than representing an immediate loss of native glands, this morphological transition reflects severe epithelial cell shrinkage, loss of apical-basal polarity, and enhanced cell sloughing. Histological studies confirmed that RhoA and ROCK are progressively upregulated in CAG and early gastric cancer tissues. Such sustained activation is closely associated with aberrant cytoskeletal remodeling and malignant transformation of the gastric mucosa [21].

#### 3.1.2. Arp2/3 Complex Hijacking and Microvillar Structural Disintegration

During the pathological progression of CAG, *H. pylori* does not directly activate RhoA via CagA alone; instead, translocated CagA initiates signaling cascades that hijack the Arp2/3 complex, the actin nucleation machinery. Under *H. pylori* infection, SRC-dependent CagA-induced signaling promotes cortactin remodeling, where cortactin functions as an adaptor protein that interacts with and stabilizes the Arp2/3 complex alongside nucleation-promoting factors such as N-WASP [22,26]. Furthermore, the Rac1/Cdc42 signaling axis is suggested to synergistically sustain this pathway [26].

Overactivation of the Arp2/3 complex leads to abnormal increases in F-actin branching, disrupting the ordered arrangement of the submembrane cortical actin network [18]. This structural perturbation potentially impairs cell polarity and epithelial integrity, exacerbating structural and functional disorders in the gastric mucosa [28]. Simultaneously, unrestrained F-actin branching depletes the intracellular free G-actin pool, limiting the supply of cytoskeletal proteins required to maintain apical microvillus integrity. This triggers microvilli effacement and loss of epithelial polarity, representing a characteristic single-cell ultrastructural alteration rather than an immediate loss of native gastric glands [28,29].

Additionally, the aberrantly activated Arp2/3 complex drives the formation of abundant lamellipodia at the leading edge of the cell, compromises cell–cell adhesion, and promotes epithelial detachment from the basement membrane. While this migratory phenotype reflects severe epithelial injury and cellular redistribution, driving full malignant transformation remains dependent on cumulative inflammatory and dynamic microenvironmental factors [30,31].

Crucially, much of the supporting evidence for this CagA–cortactin–Arp2/3 feedback loop is derived from in vitro infected-cell models rather than direct observations in gastric tissue from patients with CAG. Accordingly, these subcellular dynamics are more appropriately framed as a mechanistic hypothesis awaiting validation in clinical biospecimens.

#### 3.1.3. Inflammation and ROS Synergistically Induce Cytoskeletal Chemical Damage

In the context of persistent *H. pylori* colonization, Tinagl1 remodeling and the downregulation of epithelial defense genes synergistically foster a destructive microenvironment [32,33]. Under these conditions, epithelial damage is not solely attributable to necrosis but is also proposed to be linked to pyroptosis mediated by the virulence factor VacA. VacA activates the inflammasome via the TNFAIP3/TRAF1 axis, leading to cleavage of the pore-forming protein Gasdermin-D (GSDMD). Its activated N-terminal domain oligomerizes at the plasma membrane to form pores, directly severing the physical association between cortical actin and the cell membrane. This causes the loss of cellular structural support and triggers inflammatory lysis [34]. At the molecular level, this mechanism provides insights into how virulent factor-induced cell death contributes to loss of mucosal cellular integrity, which potentially fosters an environment conducive to progressive tissue remodeling.

In addition to virulence factor-mediated physical disruption, the persistent accumulation of reactive oxygen species (ROS) within the inflammatory microenvironment causes chemical damage to the actin cytoskeleton. These ROS primarily originate from *H. pylori* stimulation and the subsequent activation of host immune cells [35]. ROS specifically target cysteine thiol groups on actin, leading to carbonylation and aberrant disulfide bond formation, thereby inhibiting G-actin polymerization and compromising cytoskeletal repair capacity [36]. Furthermore, while oxidative stress signaling can modulate downstream actin-binding proteins such as cofilin, such regulation is inherently context-dependent and varies across distinct subcellular milieus rather than following a universal, linear dephosphorylation pathway. In the complex inflammatory landscape of chronic atrophic gastritis, fluctuating redox states can transiently tip the local balance of kinase and phosphatase activities, contributing to localized F-actin severing [7]. The combined effects cause gastric mucosal epithelial cells to progressively lose structural support during chronic infection and inflammation, contributing to cellular shrinkage and functional degeneration.

This persistent exogenous damage forces epithelial cells to initiate energy-intensive cytoskeletal repair processes. However, the dynamic reconstruction of the cytoskeleton is highly dependent on the mitochondrial ATP supply, meaning that exogenous disruption potentially depletes bioenergetic reserves and induces a deeper endogenous metabolic crisis.

#### 3.1.4. Toll-like Receptors and MicroRNAs in Pathogen Persistence

The progressive loss of native gastric glands requires persistent *H. pylori* colonization to maintain chronic microenvironmental stress. This persistence is significantly facilitated by the subversion of host mucosal immune surveillance. Specifically, host Toll-like receptors (TLRs) are continuously engaged by pathogen-associated molecular patterns, which can induce localized immune tolerance and prevent effective bacterial clearance [37]. Concurrently, the dysregulation of host microRNAs (miRNAs) during infection further contributes to immune evasion and sustains persistent mucosal inflammation [38]. This TLR- and miRNA-mediated immune tolerance enables long-term pathogen survival, thereby providing the continuous inflammatory stimuli required to drive the aforementioned cytoskeletal disruption and subsequent mitochondrial dysfunction.

### 3.2. Endogenous Drivers: Mitochondrial Bioenergetic Impairment and Cytoskeletal Remodeling Failure

During CAG progression, the fundamental pathological hallmark of gastric mucosal epithelial cells is chronic bioenergetic deficiency. In this state, persistent insufficient mitochondrial ATP production inhibits the dynamic remodeling of the energy-intensive actin cytoskeletal system, depriving it of its self-repair capacity and fostering the progressive degeneration of epithelial and glandular structures [39]. Therefore, the functional decoupling of mitochondria and the cytoskeleton is proposed as a core pathological pathway linking endogenous metabolic impairment to the progressive loss of native gastric glands.

#### 3.2.1. Mitochondrial Quality Controls Dysregulation and ATP Deficiency

In experimental and pathological models of chronic atrophic gastritis, the mitochondrial quality control (MQC) system and cellular bioenergetic networks are profoundly disrupted. Persistent pathogenic factors, such as *Helicobacter pylori* infection, directly trigger mitochondrial stress in gastric epithelial cells. This metabolic perturbation leads to prominent structural abnormalities, including mitochondrial swelling and cristae fragmentation, which reflect a severe loss of mitochondrial integrity [36,40]. This alteration reduces the efficiency of mitochondrial oxidative phosphorylation, leading to inadequate bioenergy production.

As a conserved cellular mechanism, ATP plays a fundamental role in F-actin polymerization and dynamic stability; its depletion can compromise G-actin nucleation and the maintenance of filamentous structures [41]. In the context of CAG, insufficient ATP production is hypothesized to be a key intrinsic contributor to the destabilization of the gastric F-actin cytoskeletal network. However, given the metabolic adaptability of epithelial cells, bioenergetic impairment alone acts in concert with other stress signals rather than as an isolated driver. Mitochondrial dysfunction has been demonstrated to be significantly correlated with the progression of gastric mucosal atrophy [36]. Furthermore, impaired energy metabolism is often accompanied by redox imbalance, which further exacerbates dysregulated cytoskeletal remodeling, establishing a pathological cycle in which energy deficiency and structural damage mutually reinforce each other [42,43].

#### 3.2.2. Abnormal Cofilin Activation by mtROS Drives Actin Filament Disassembly

The stability of the F-actin cytoskeleton is crucial for maintaining the structure and function of the gastric glands. Mitochondrial dysfunction is proposed to destabilize the F-actin network through mtROS signaling, leading to loss of epithelial cell polarity and impaired secretory function [36]. In the context of MQC dysfunction, mtROS primarily disrupt cytoskeletal homeostasis through multilevel mechanisms.

First, as established in broad cellular models, cofilin is a core regulator of cytoskeletal dynamics. Under physiological conditions, cofilin activity is strictly regulated via phosphorylation/dephosphorylation cycles. Rather than acting via a universal pathway, mtROS can perturb the upstream LIMK/SSH signaling axis through oxidative modifications, with its impact on cofilin activation being highly context- and stress-dependent [44]. Under conditions of insufficient ATP supply, the depolymerized actin network fails to effectively reassemble, ultimately leading to network collapse.

Second, dysbiosis of the gastric microbiota and metabolic profiles in CAG patients are significant contributing factors to increased oxidative stress [45]. Concurrently, progressive gastric mucosal injury and chronic inflammation challenge endogenous cytoprotective mechanisms, where the compensatory failure or impairment of antioxidant defense systems, such as the Nrf2/HO-1 signaling axis, exacerbates redox-mediated cellular damage and drives the progression of chronic atrophic gastritis [46].

Third, drawing from foundational cell biology, the suppression of cytoskeletal dynamics itself can paradoxically exacerbate tissue homeostatic imbalance. Targeted inhibition of actin polymerization disrupts epithelial homeostasis [47,48]. This mechanism suggests that impaired cytoskeletal remodeling due to mitochondrial damage is not only a pathological consequence but also a potential key driver contributing to the progressive loss of native gastric glands.

#### 3.2.3. Adherens and Tight Junction Dissociation and Loss of Native Gastric Glands Mediated by Mechanotransduction Failure

The breakdown of the mitochondrial–cytoskeletal coupling is proposed to manifest as mechanotransduction failure, leading to the dissociation of intercellular junctions and contributing to the progressive loss of native gastric glands. In fundamental cellular models, α-catenin serves as a critical mechanosensitive anchor linking F-actin to adherens junctions (AJs, via E-cadherin). Research indicates that the stability of α-catenin–F-actin interactions depends on mechanical tension, which results in the formation of “catch bonds” under mechanical homeostasis to maintain the connection. When F-actin breaks and tension is lost, α-catenin transitions to “slip bonds,” destabilizing AJs [49,50]. We hypothesize that during CAG pathogenesis, this mechanotransduction failure indirectly drives the dissociation of tight junction (TJ) components (such as ZO-1/Occludin) from the cell membrane.

**Figure 1:**
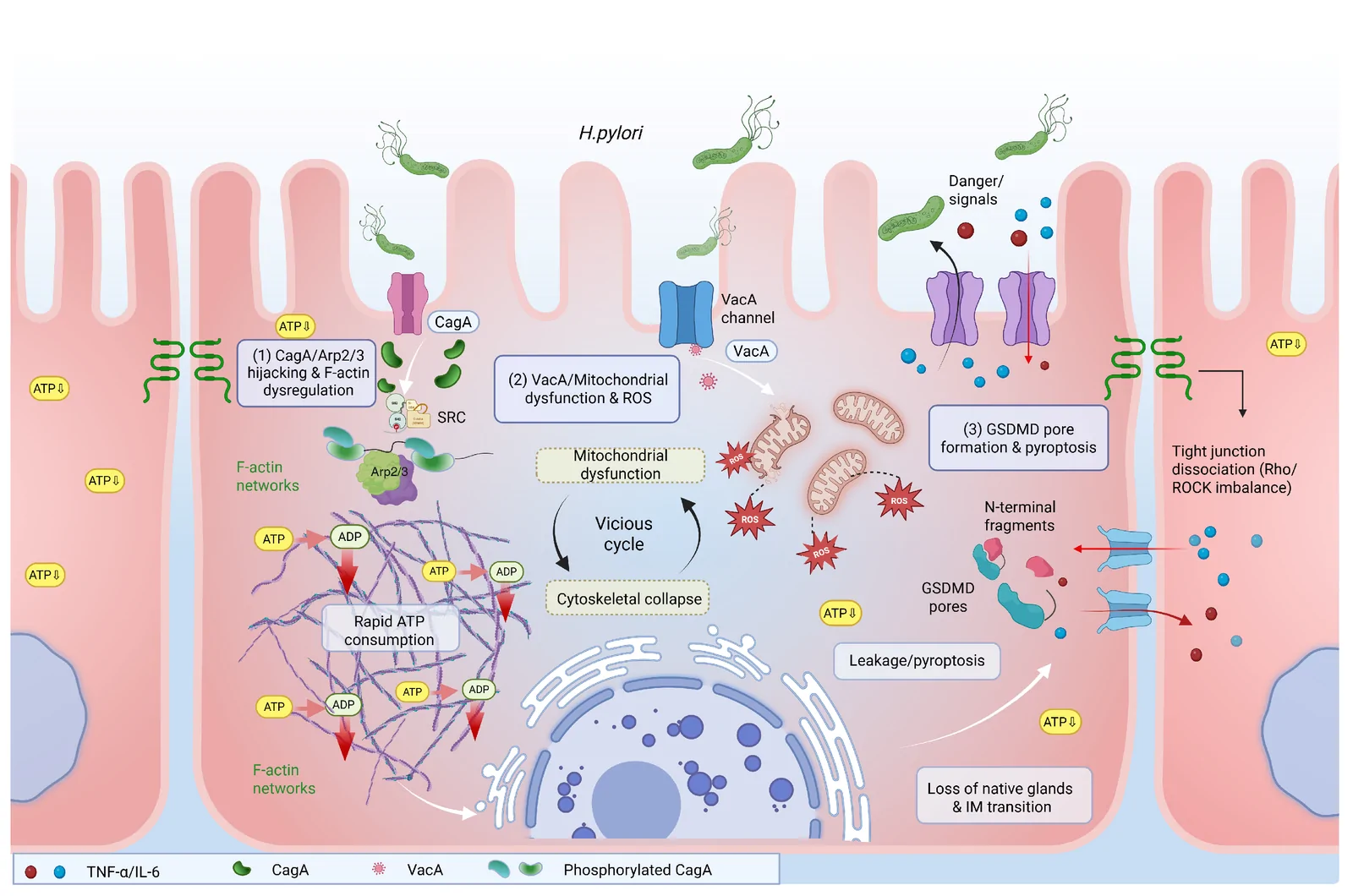
Schematic of *H. pylori*-mediated actin collapse, mitochondrial dysfunction and pyroptosis. (Note: The causal relationships depicted here represent a hypothesized mechanistic framework based on experimental models, which have not yet been definitively demonstrated by primary in vivo studies).

Single-cell sequencing confirmed that CAG patients exhibit significantly downregulated expression of actin and claudin family genes in atrophic mucosa, suggesting concurrent impairment of both epithelial barrier structure and mechanical homeostasis [33]. This mitochondrial dysfunction-driven collapse of the cytoskeleton and dissociation of junctional complexes potentially leads to loss of cell polarity and epithelial sloughing, which further fosters the progressive loss of native gastric glands, forming a “mitochondrial–cytoskeletal–glandular atrophy” cascade [51] (Figure 1).

## 4. From Barrier Disruption to Malignant Invasion: The Inflammation-Cancer Cascade Triggered by Actin Cytoskeletal Dysregulation

The transition from atrophic gastritis (CAG) to gastric cancer (GC) involves not only an accumulation of inflammation but also a progressive pathological process characterized by an imbalance in glandular structure and mechanical homeostasis. Within this context, damage to the actin cytoskeleton is proposed to disrupt mucosal barrier and glandular functions, thereby promoting inflammation-associated carcinogenesis through epithelial–mesenchymal transition (EMT) and microenvironmental remodeling [14]. In fundamental biophysical models, cofilin-mediated cytoskeletal softening and dysregulated crosslinking profoundly alter cellular biomechanics [52]. We hypothesize that within the gastric mucosa, such excessive biomechanical dysregulation accelerates structural instability, potentially contributing to subsequent malignant progression during CAG [30].

### 4.1. Barrier Instability and Glandular Structural Disintegration: The Pathological Origin of Cytoskeletal Damage

In the early stages of CAG progression, the integrity of the actin cytoskeleton serves as the primary support for the epithelial barrier. As demonstrated in fundamental epithelial models, the F-actin network forms a structural cytoskeleton that maintains the steady-state integrity of tight junction complexes, particularly ZO-1 and Occludin, ensuring the mechanical resilience of apical junctions [53]. Consequently, we propose that the disruption of this cytoskeletal framework reduces this resilience, rendering the mucosal barrier highly fragile and hyperpermeable. Barrier breakdown allows the gastric microbiota and its metabolites to infiltrate the mucosal layer, resulting in dysregulated local metabolic profiles and an exacerbated inflammatory burden, which further disrupts microenvironmental homeostasis [45].

Under the dual pressures of cytoskeletal support loss and downregulation of the defense system, gastric epithelial cells are compelled to initiate adaptive phenotypic remodeling. Intestinal metaplasia (IM), the pivotal node in the transformation of CAG to gastric cancer, represents a pathological compensatory mechanism driven by molecular reprogramming and downregulation of epithelial defense systems [2,54], a process hypothesized to be exacerbated by the concurrent loss of cytoskeletal support. Although pure pseudopyloric metaplasia (PPM) in the gastric corpus lacks direct carcinogenicity, it represents a reparative pathological change occurring in the context of CAG and indicates profound disruption of the mucosal microenvironment and cytoskeletal homeostasis [33,55]. Therefore, disruption of the barrier and cytoskeletal homeostasis not only compromises surface defense mechanisms but also establishes the structural foundation for the disintegration of glandular cell polarity and the collapse of glandular secretory function, thereby propelling the mucosal deterioration toward the progressive loss of native gastric glands.

### 4.2. Loss of Polarity and Disintegration of the Functional Axis

The loss of native gastric glands involves not only a reduction in or disappearance of the gastric glands but also disintegration of the tissue structure and functional axes. The actin cytoskeleton is crucial for maintaining apical–basal polarity and coordinated secretion in gastric gland cells. Its disruption leads to the mislocalization of polarity proteins and key tight junction components characteristic of the gastric mucosa, such as Claudin-18, thereby compromising glandular structural integrity [4,56]. In the context of impaired cytoskeletal homeostasis, inflammation-induced pyroptosis accelerates the loss of parietal and chief cells, exacerbating progressive glandular degeneration [57]. More critically, cytoskeletal abnormalities can disrupt vesicular transport and the targeted release of secretory proteins, impairing proton pump vesicular trafficking and the efficiency of gastric acid secretion in parietal cells [11]. This structural collapse and subsequent loss of functional parietal cells profoundly alter the mucosal microenvironment. Consequently, residual epithelial cells are driven into a state of functional decline characterized by extensive dedifferentiation [58]. These dedifferentiated cells, having lost their mature secretory characteristics and exhibiting polarity disruption, enter a state of extreme cellular plasticity, a condition that represents a critical structural predisposition for subsequent malignant transformation [15,16].

### 4.3. Actin Cytoskeletal Rearrangement Drives the Gastric Inflammation-to-Carcinoma Transition

Glandular atrophy is a pivotal step in the Correa cascade of inflammation-to-carcinoma progression [59]. Within this sequence, cytoskeletal remodeling is hypothesized to drive further cellular transformation. This structural alteration is directly supported by evidence from early *H. pylori* infection and intestinal metaplasia [18,60]. As the core inducer of CAG, *H. pylori* infection disrupts mucosal homeostasis by stimulating macrophages to secrete CCL3 [61]. It also promotes the formation of neutrophil extracellular traps (NETs), a process fundamentally dependent on actin cytoskeleton rearrangements [62]. These events collectively establish an early procarcinogenic microenvironment. In a persistent inflammatory environment, immune cell infiltration and ROS release drive nuclear F-actin polymerization, a mechanism amplifying DNA damage and disrupting cytoskeletal integrity [63].

Thus, the disruption of normal cytoskeletal mechanosensing subverts epithelial homeostasis. This impairment confers aberrant tolerance to adhesion-triggered cell death in dedifferentiated cells. Consequently, early nascent clones can survive and expand within architecturally disrupted mucosal tissues, driving their progression toward dysplasia [30,51]. At this stage, Claudin-18.2 is highly heterogeneously expressed, reflecting not only the degree of disruption in the cytoskeletal–junction linkage but also the invasive phenotype and poor prognosis of the tumor [64,65]. CRISPR/Cas9 organoid models confirmed that the absence of the actin-stabilizing protein cortactin significantly inhibits the malignant transformation of dysplastic cells into adenocarcinoma cells, directly establishing the pivotal role of cytoskeletal stability in blocking gastric cancer development [30].

**Figure 2:**
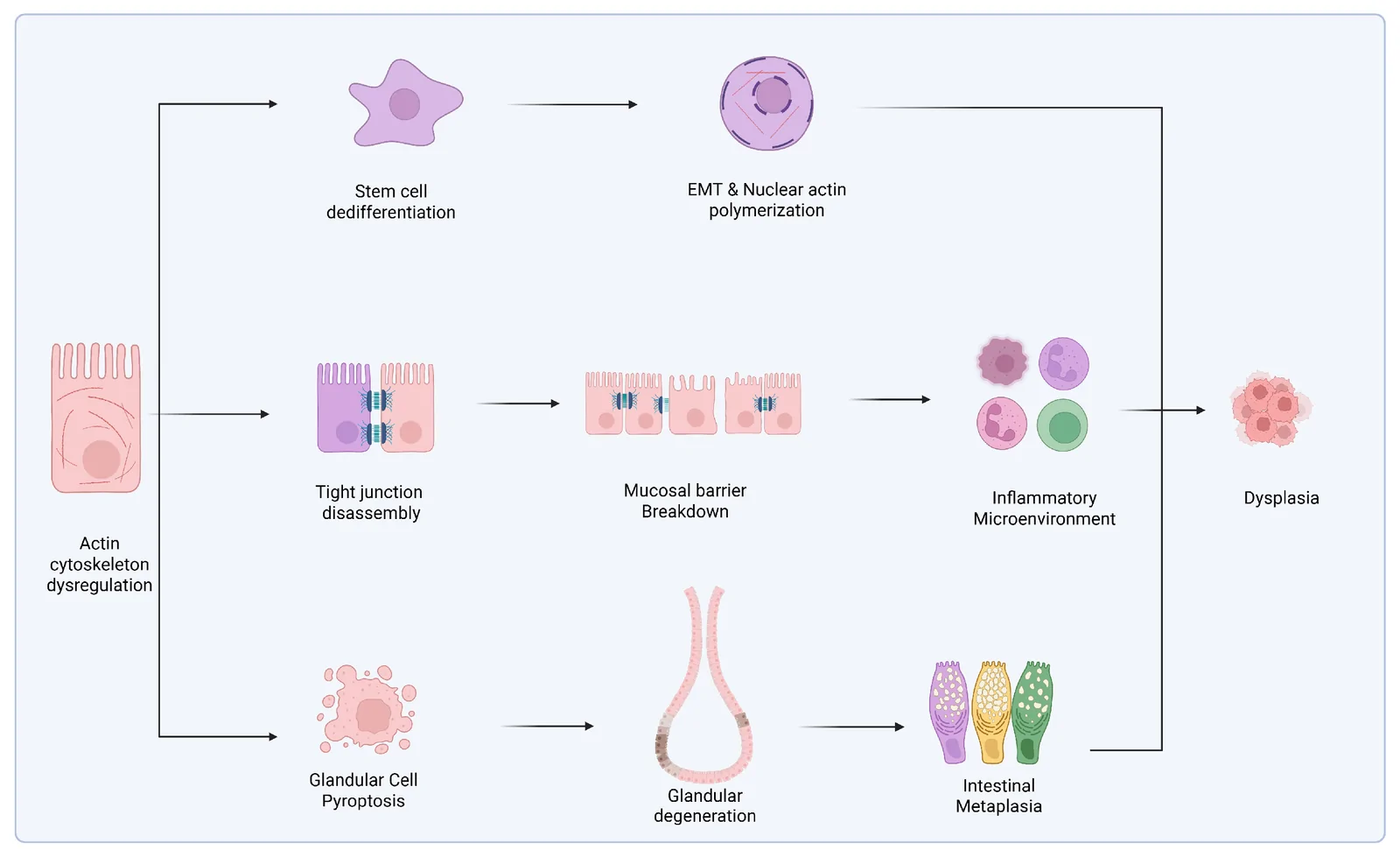
Pathological chain of actin cytoskeleton disruption-driven oncogenic transformation in chronic atrophic gastritis. (Note: This illustrated progression represents a proposed theoretical model rather than a definitively established clinical pathway).

In the context of glandular atrophy, the survival advantage conferred by actin cytoskeletal remodeling, coupled with a systemic shift in gastric stem cell dynamics, is increasingly recognized as a major contributor to inflammation-driven carcinogenesis [51] (Figure 2).

## 5. Exploratory Therapeutic Implications: Putative Intervention Strategies Targeting the “Energy–Cytoskeleton” Axis

### 5.1. Ameliorating Glandular Degeneration and Blocking Cancer Progression: Exploring Potential Molecular Targets on the “Energy-Cytoskeleton” Axis

Precision interventions targeting mitochondrial dysfunction and cytoskeletal disorders may offer novel entry points for ameliorating CAG-associated glandular degeneration and impeding the inflammation–carcinoma transition (Table 1):

At the level of glandular regeneration, stem cell activation and cytoskeletal remodeling are key. Gastric glandular regeneration relies on the activation of gastric stem cells (GSCs) and the physical support provided by their niche. Research suggests that activating GSCs and remodeling their surrounding cytoskeletal networks potentially assists in the restoration of glandular architecture and mitigate glandular loss-related phenotypes [67].

At the level of epithelial homeostasis, maintaining tight junction homeostasis is crucial. Claudin-18.2 is a key molecule sustaining the integrity of tight junctions in the gastric mucosal epithelium. Its abnormal exposure indicates loss of epithelial polarity and structural instability and has been demonstrated to be significantly correlated with the carcinogenic process [56]. Intervention strategies targeting this molecule provide insights into inflammation-driven carcinogenesis from the perspectives of junctional homeostasis and cytoskeletal remodeling [66].

At the level of malignant transformation, blocking aberrant cytoskeletal reorganization may be an effective strategy. EMT-related cytoskeletal remodeling is pivotal in the progression from glandular epithelium to carcinoma. Modulating EMT signaling or cytoskeletal dynamics can theoretically reduce the risk of malignant progression [68].

These findings suggest that targeting signaling and structural homeostasis along the “energy–cytoskeleton” axis holds promise as a potential strategy against CAG inflammation-driven carcinogenesis.

### 5.2. Exploratory Pharmacological Remodeling Strategies Targeting Mitochondria–Cytoskeleton Coupling

Pharmacological interventions targeting the pathological mechanism of “mitochondria–cytoskeleton coupling” are hypothesized to rely on the dual repair mechanisms of energy metabolism reprogramming and microenvironmental matrix remodeling.

First, mitochondrial-derived ATP deficiency lies at the core of abnormal F-actin polymerization. Emerging pharmacological evidence suggests that certain plant-derived metabolic modulators can mitigate gastric mucosal injury by targeting mitochondrial dysfunction [69]. Within our mechanistic framework, this metabolic restoration is proposed to provide the bioenergetic foundation required for F-actin nucleation and polymerization, thereby potentially contributing to the repair of the gastric mucosal barrier in CAG [39,69].

Second, to address the matrix stiffening and EMT tendencies resulting from the transition from late-stage CAG chronic inflammation to interstitial fibrosis, therapeutic efforts should prioritize the holistic modulation of abnormal cytoskeletal remodeling, rather than relying on non-specific chemical inhibitors that may induce systemic toxicity. Multicomponent herbal formulations (such as Moluodan) exhibit the potential to modulate inflammatory signaling networks through multitarget mechanisms, potentially contributing to the suppression of pathological cytoskeletal reorganization during the progressive loss of native gastric glands and thereby impeding the inflammation-to-carcinoma transition [70].

### 5.3. A Tiered Prevention and Treatment Pathway Targeting Inflammation-Driven Carcinogenesis

At the mechanistic level, chronic atrophic gastritis at different stages of disease progression corresponds to varying degrees of cytoskeletal damage and repair capacity. This characteristic provides a conceptual biological foundation for exploring stage specific, target-specific intervention strategies.

Primary prevention (*H. pylori* infection phase): Aims to protect the cytoskeleton framework and mucosal barrier while reducing the inflammatory burden. By eradicating *H. pylori* and supplementing it with microenvironmental regulation strategies, inflammatory damage to the physical structure of the cytoskeleton framework may be minimized [71,72].

Secondary Prevention (Atrophic and Intestinal Metaplasia Stage): Focuses on stabilizing the microenvironment and cell junctions to potentially delay glandular degeneration. Emerging evidence suggests that targeting the regulation of lipid metabolism and tight junction-associated molecules (e.g., claudin-18.2) could theoretically aid in maintaining glandular structures [56].

**Figure 3:**
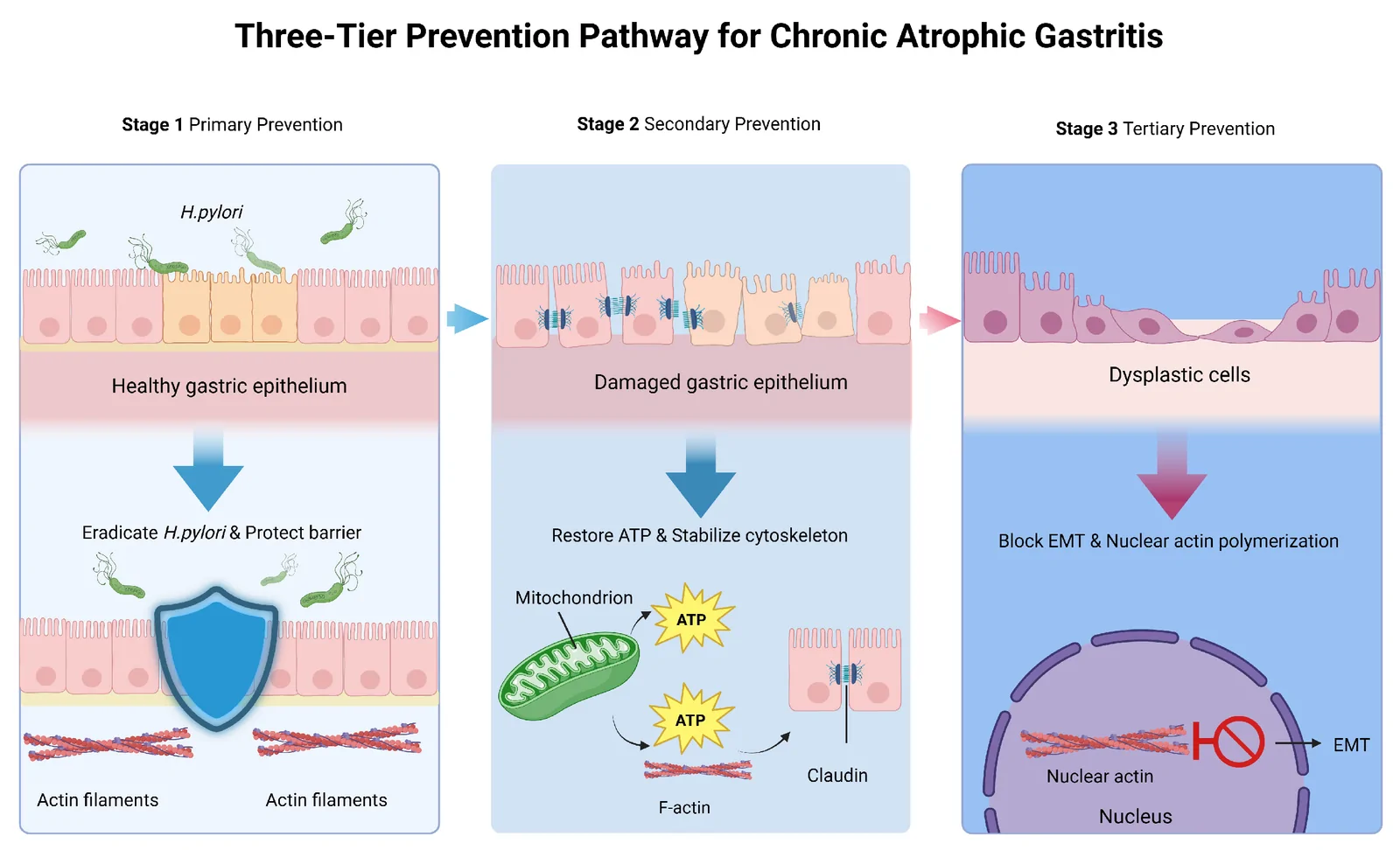
Three-Tier Prevention Strategy for CAG: The Energy–Cytoskeleton Axis (Note: These exploratory interventions are based on preclinical hypotheses and remain to be incorporated into current clinical guidance).

Tertiary Prevention (Intraepithelial Neoplasia Stage): Explores the blockade of nuclear actin remodeling as a conceptual strategy to halt EMT and malignant transformation. As demonstrated in fundamental cellular models, nuclear actin polymerization is a key mechanism driving EMT [73], a process critical for the progression from precancerous lesions to invasive carcinoma. In addition to endoscopic surveillance in accordance with the European Society of Gastrointestinal Endoscopy (ESGE) MAPS guidelines [74], future research may explore natural bioactive molecules that specifically inhibit aberrant nuclear actin polymerization as an experimental strategy to prevent progression to invasive gastric carcinoma (Figure 3).

## 6. Conclusions

This review proposes a comprehensive mechanistic framework suggesting that the decoupling of mitochondrial bioenergetics and actin cytoskeletal dynamics plays a fundamental role in driving the progressive loss of native gastric glands in CAG. When initiated by *Helicobacter pylori* infection and chronic inflammation, mitochondrial ATP depletion and cytoskeletal destabilization are proposed to disrupt epithelial polarity and mechanotransduction, thereby contributing to glandular degeneration and carcinogenesis.

While this synthesis relies primarily on in vitro and multi-omics evidence rather than direct in vivo validation, these insights indicate that conventional anti-inflammatory and antibacterial strategies can significantly benefit from restoring “energy–cytoskeleton” homeostasis. Future studies utilizing animal models and patient-derived organoids will be essential to validate these coupling pathways, ultimately offering promising opportunities for therapeutic intervention in CAG.

## Figures and Tables

**Table 1:** Key molecular drivers and pathological consequences of cytoskeletal dysregulation in CAG.

Category	Key Molecule	Putative Mechanism in CAG	Impact on Cytoskeleton	Refs.
Exogenous Factors	CagA/VacA	Hijacking Arp2/3; triggering pyroptosis +1	Microvillar effacement; cortical ring collapse +1	[26,28,34]
Signaling Axis	RhoA/ROCK	Aberrant activation by *H. pylori*	Disrupted mechanosensing; junctional dissociation	[21]
Energy Supply	ATP/mtROS	Mitochondrial bioenergetic impairment	Inhibition of F-actin nucleation and extension +1	[36,39]
Dynamic Tuning	Cofilin	Dephosphorylation by oxidative stress	Excessive filament cleavage and depolymerization	[30,44,52]
Barrier Integrity	Claudin-18.2	Loss of epithelial polarity	Disruption of the cytoskeleton-junction linkage	[56,66]

## Data Availability

No new data were created or analyzed in this study. Data sharing is not applicable to this article.

## Abbreviations

The following abbreviations are used in this manuscript:

CAG	chronic atrophic gastritis
*H. pylori*	Helicobacter pylori
ATP	adenosine triphosphate
ROS	reactive oxygen species
EMT	epithelial–mesenchymal transition
ZO-1	zonula occludens-1
RhoA	Ras homolog family member A
Rac1	Ras-related C3 botulinum toxin substrate 1
Cdc42	cell division cycle 42
ROCK	Rho-associated protein kinase
Abl	Abelson tyrosine kinase
Arp2/3	actin-related proteins 2/3
LIMK	LIM domain kinase
SSH	slingshot protein phosphatase
TNF-α	tumor necrosis factor-alpha
IL-6	interleukin-6
CagA	cytotoxin-associated gene A
TRAF	TNF receptor-associated factor
TNFAIP3	TNF alpha-induced protein 3
ARPC2	actin related protein 2/3 complex subunit 2
CCL3	C-C motif chemokine ligand 3
CRISPR/Cas9	clustered regularly interspaced short palindromic repeats/CRISPR-associated protein 9
